# Supplementary material for: Identification and characterization of tryptophan metabolism-related genes in carotid artery plaques
Source: PLoS One. 2026 Feb 2;21(2):e0341122. doi: 10.1371/journal.pone.0341122 (PMC12863520; doi:10.1371/journal.pone.0341122)
Supplement: Attachment — Submitted filename: Response to Reviewers.docx [file pone.0341122.s007.docx]

**Academic editor**

**1.The absence of a duplicate dataset undermines the study's conclusion and raises question about the reliability. Additional experiments involving a duplicate dataset are required. In addition, the authors should indicate what the strengths and limitations of their findings are.**

Thank you for your suggestion. We fully recognize that external cohort validation will further enhance the robustness of the research results. However, the currently available carotid plaque RNA-seq cohorts are very limited, and we are unable to obtain reliable external datasets. In addition, obtaining new clinical plaque specimens requires ethical approval and sample collection, which is beyond the scope and time of current transcriptomic research. To make up for the absence of duplicate datasets, we enhance the biological rationality by incorporating mechanical evidence from the literature. In addition, we added a dedicated section in the discussion to address the advantages and limitations, explicitly acknowledging the lack of an external validation queue and clarifying that future work will include validation in independent datasets and experimental models. We believe that through these revisions, this research remains scientifically rigorous and provides valuable biological insights as a computational discovery study.

In lines 361-371:

In this study, we identified multiple TRDEGs (such as TDO2, KMO and KYNU) that jointly point to the abnormal activation of the tryptophan kynuurine pathway, which is the main direction of tryptophan catabolic metabolism and has been continuously confirmed in recent years to be closely related to the occurrence and development of atherosclerosis. In human atherosclerotic plaques, IDO1 is significantly upregulated in macrophage-rich regions and is associated with plaque instability by enhancing kyurine metabolism and promoting the expression of local tissue factors^[36]^ . In line with human studies, animal experiments have further confirmed that when the IDO1-kyrie acid axis is disrupted, it accelerates vascular calcification and plaque formation. Exogenous supplementation of kynurenine can reverse the above adverse changes through immune metabolic signal regulation^[31]^. These pieces of evidence collectively indicate that the disorder of tryptophan metabolism is a key regulatory link closely coupled with the remodeling of the immune microenvironment of carotid plaques.

In lines 380-384:

This study provides a multi-dimensional transcriptomic analysis integrating bulk RNA-seq, single-cell data, and immune infiltration profiling. However, due to the limited availability of comparable public carotid plaque datasets, external cohort validation was not performed. Therefore, the findings should be interpreted as hypothesis-generating, and future studies with larger independent cohorts and experimental validation are warranted.

**Reviewer #1**

**1.The full names of Kyn and 5-HT in line 55, as well as TDO and IDO in line 57, are missing.**

Thank you for your suggestion, we added the full names of Kyn, 5-HT, TDO and IDO in lines 52 to 57 in the revised manuscript.

In lines 52 to 57:

Its levels depend both on dietary intake and the activity of three main metabolic pathways: the kynurenine (Kyn), 5-hydroxytryptamine (5-HT), and indole pathways^[5]^ .The kynurenine pathway, responsible for over 95% of tryptophan degradation, is regulated by the rate-limiting enzymes TDO (Tryptophan 2,3-dioxygenase), IDO1 (Indoleamine 2,3-dioxygenase 1), and IDO2 (Indoleamine 2,3-dioxygenase 2)^[6]^.

**2.The second paragraph (introducing the three tryptophan metabolism pathways) and the third paragraph (describing their relationships to carotid artery plaques) could be combined to provide a more concise rationale.**

Thank you for your suggestion, we combined the second and third paragraphs of the Introduction to provide a more concise rationale.

In lines 50 to 71:

Tryptophan is an essential amino acid that the human body cannot synthesize and must be obtained through diet. Beyond serving as a building block for proteins, tryptophan participates in neurotransmitter synthesis, immune regulation, and the modulation of sleep and mood. Its levels depend both on dietary intake and the activity of three main metabolic pathways: the kynurenine (Kyn), 5-hydroxytryptamine (5-HT), and indole pathways^[5]^ .The kynurenine pathway, responsible for over 95% of tryptophan degradation, is regulated by the rate-limiting enzymes TDO (Tryptophan 2,3-dioxygenase), IDO1 (Indoleamine 2,3-dioxygenase 1), and IDO2 (Indoleamine 2,3-dioxygenase 2)^[6]^. This pathway generates kynurenine and related metabolites that modulate inflammation, immune responses, and excitatory neural signaling, and have been linked to various diseases. Alterations in kynurenine pathway activity are associated with carotid plaque formation and instability: Munn et al. reported that changes in key enzyme activity correlate with plaque instability^[7]^, while Shen et al. demonstrated that IDO1 influences atherosclerosis via local inflammation and immune regulation^[8]^. Kynurenine metabolites may also affect endothelial function, further contributing to plaque development. The 5-HT pathway converts tryptophan into 5-hydroxytryptophan (5-HTP) and subsequently into 5-hydroxytryptamine (serotonin)^[9]^. While 5-HT functions primarily as a neurotransmitter, it also impacts vascular endothelium and platelets, potentially promoting plaque formation through regulation of vascular tone, smooth muscle proliferation, and platelet activation. The precise contribution of serotonin to carotid atherosclerosis remains under investigation. Finally, the indole pathway, mediated by gut microbiota, produces indole and its derivatives, which help maintain intestinal homeostasis, modulate systemic inflammation, and regulate immune responses^[10]^. Given that atherosclerosis is part of the systemic inflammatory response, indole metabolites may influence plaque formation and stability by reducing inflammation and protecting vascular endothelium^[11,12]^.

**3.The language is generally clear, but there are several grammar mistakes, and a few long sentences should be shortened to improve readability.**

Thank you for your suggestion, we revised the manuscript to correct grammatical errors and have shortened overly long sentences to improve clarity and readability.

**4.TPH1, MAOB, TDO2, KMO, KYNU, and CYP1B1 were identified as tryptophan metabolism–related genes involved in carotid plaque progression, and their potential mechanisms are well discussed. Including a schematic figure at the end to illustrate the proposed mechanisms would further strengthen the manuscript and help readers grasp the main findings.**

Thank you for your suggestion, at the end of the article, we added a schematic figure to illustrate the proposed mechanisms, and in the discussion, we optimized our presentation of the mechanisms based on the schematic figure.

In lines 354-360:

Meanwhile, MAOB is a 5-HT degrading enzyme and belongs to the downstream regulatory factor of the tryptophan - serotonin branch^[33]^. However, the reduction of the tryptophan - serotonin metabolic pathway may have affected the expression of MAOB, but the specific regulatory direction remains to be further determined. Finally, kynurenine can also act as an endogenous AhR agonist^[34]^. CYP1B1 (Cytochrome P450 1B1) is a downstream target gene of AHR and is involved in the metabolism of cholesterol and other lipids^[35]^, and these lipids are key components in the occurrence of atherosclerosis (Fig.6).

In lines 392-400:

**Fig 6. The proposed mechanisms linking TRDEGs to immune dysregulation and plaque progression in carotid atherosclerosis.** This schematic illustrates how six key TRDEGs coordinate metabolic and immune alterations in carotid atherosclerotic plaques. Up-regulated kynurenine pathway enzymes (TDO2, KMO and KYNU) enhance kynurenine production, and activate the AhR signaling pathway. Meanwhile, serotonin synthesis is reduced, accompanied by decreased smooth muscle cells, while MAOB-mediated 5-HT degradation promotes oxidative stress and endothelial dysfunction. In parallel, CYP1B1 participates in AhR-dependent lipid metabolic reprogramming, further aggravating plaque formation. Together, these TRDEG-driven metabolic changes disrupt vascular homeostasis and accelerate the progression of carotid artery plaques.

**Reviewer #2**

**1.The study mainly analyzed the correlation between tryptophan metabolism related genes and atherosclerotic lesions.**

**The study's primary objective, as indicated by the title, was to identify tryptophan metabolism-related differential genes in atherosclerotic plaques. However, the identification of differential genes relied solely on a single RNAseq dataset without validation. While enrichment analysis was conducted for these genes, the p-value for tryptophan metabolism pathway enrichment reached 0.05. Moreover, the combined analysis of upregulated and downregulated genes failed to clarify whether tryptophan metabolism pathways were downregulated or upregulated during disease progression. Although logistic regression models attempted to demonstrate correlations between tryptophan metabolism-related differential genes and atherosclerosis, the small sample size resulted in an AUC value of merely 0.75. Correlation analyses aimed to establish links between these differential genes and plaque immune cell infiltration phenotypes, but neither immune infiltration phenotypes were validated in single-cell RNAseq data nor did they undergo multiple hypothesis correction. Finally, six amino acid metabolism-related genes showed differential expression across cell types in the single-cell RNAseq dataset. However, no cell type clustering markers were provided, and the absence of validation using known marker gene expression profiles (e.g., CD68 for macrophages) may have introduced bias in cell clustering.**

Thank you for your suggestion, we will answer your questions in several ways: (1) Since no other external datasets that meet the conditions were collected, the verification of external datasets does not appear in the article. (2) To demonstrate the tryptophan metabolic pathway, we added GSEA analysis to elaborate on the upregulation of the pathway. The results show that the tryptophan metabolic pathway is differentially regulated in carotid plaques, and this result may be limited by the scale of our data. However, its expression trend in carotid artery plaques is upregulated, suggesting that the tryptophan metabolic pathway may be overexpressed in carotid artery plaques. (3) As in (1), the lack of external data prevents us from verifying and improving the model. (4) We added multiple hypothesis validations of the correlation between tryptophan-related genes and immune infiltration phenotypes and updated them in the manuscript and Figure 3C. (5) We added the expression of markers for each single-cell type to verify our single-cell annotation results.

In lines 169-174:

Although the P-value of the enrichment analysis for the tryptophan metabolic pathway did not reach statistical significance (p.adj.value = 0.97), based on the standardized enrichment score (NES), it could be observed that this pathway showed an upregulation trend in plaque tissues (NES = 0.54). This trend suggests that although the sample size is limited and the statistical significance is insufficient, tryptophan-related genes may be in an activated state as a whole in atherosclerotic plaques.

In lines 254-256:

(C) Heatmap of correlations between TRDEGs’ expression and immune cell infiltration proportions. Correlation analysis was performed using the Spearman rank correlation method. * p.adj < 0.05, ** p.adj < 0.01, and *** p.adj < 0.001.

In lines 321-322:

(C) Expression patterns of canonical marker genes across annotated cell types in the single-cell dataset.

**2.The data are insufficient to support the conclusion:**

**Regarding the identification of differentially expressed genes, the study utilized only a single RANseq dataset, lacking duplicate validation. To ensure reliability, at least two independent human cohort RNAseq datasets should be added as validation sets for repeated verification. Alternatively, differential genes could be validated using either a single human cohort RNAseq dataset or multiple atherosclerotic mouse model RNAseq datasets. Furthermore, RNAseq results can be cross-validated through qPCR, Western blotting, or immunohistochemistry in human or mouse samples. Whether tryptophan metabolic pathways are upregulated or downregulated in disease states warrants investigation. We recommend conducting GSEA enrichment analysis on tryptophan metabolic pathways to confirm this hypothesis.**

**It is suggested to use in vitro cell models (such as vascular endothelial cells) to verify the effects of TRDEGs on inflammation and lipid metabolism.**

**Why not validate the aforementioned immune cell infiltration with single-cell RANseq data? Single-cell RNAseq requires the provision of markers to distinguish each cell type, and it needs to be validated using recognized cell-type markers.**

**It is recommended to provide analytical code to ensure the reproducibility of the results.**

Thank you for your suggestion, we will answer your questions in several ways: (1) Due to the lack of a suitable bulk dataset of carotid artery plaques, we were unable to add data to repeatedly verify the differentially expressed genes. (2) We sincerely thank the reviewers for suggesting additional experimental verification (such as qPCR, Western blotting or immunohistochemistry). Although we fully agree that wet laboratory validation will further strengthen the conclusion, experimental validation requires obtaining fresh human carotid plaque specimens and corresponding ethical approvals, which are not available within the current research scope and time frame. Furthermore, carotid plaque biology shows significant heterogeneity among patients, and small-sample validation does not necessarily provide more convincing evidence than multi-dataset computational support. Therefore, we have decided not to include wet chamber verification in this version of the manuscript. To make up for this, we have added mechanism support for the association between TRDEGs and tryptophan metabolism and atherosclerosis on the basis of the literature, and will further verify it in future experimental studies. (3)To demonstrate the tryptophan metabolic pathway, we added GSEA analysis to elaborate on the upregulation of the pathway. The results show that the tryptophan metabolic pathway is differentially regulated in carotid plaques, and this result may be limited by the scale of our data. However, its expression trend in carotid artery plaques is upregulated, suggesting that the tryptophan metabolic pathway may be overexpressed in carotid artery plaques. (4) All the analysis scripts used in this study have been stored on GitHub. The repository will be publicly accessible and a corresponding link will be provided when accepting this manuscript.

In lines 361-371:

In this study, we identified multiple TRDEGs (such as TDO2, KMO and KYNU) that jointly point to the abnormal activation of the tryptophan kynuurine pathway, which is the main direction of tryptophan catabolic metabolism and has been continuously confirmed in recent years to be closely related to the occurrence and development of atherosclerosis. In human atherosclerotic plaques, IDO1 is significantly upregulated in macrophage-rich regions and is associated with plaque instability by enhancing kyurine metabolism and promoting the expression of local tissue factors^[36]^ . In line with human studies, animal experiments have further confirmed that when the IDO1-kyrie acid axis is disrupted, it accelerates vascular calcification and plaque formation. Exogenous supplementation of kynurenine can reverse the above adverse changes through immune metabolic signal regulation^[31]^. These pieces of evidence collectively indicate that the disorder of tryptophan metabolism is a key regulatory link closely coupled with the remodeling of the immune microenvironment of carotid plaques.

In lines 169-174:

Although the P-value of the enrichment analysis for the tryptophan metabolic pathway did not reach statistical significance (p.adj.value = 0.97), based on the standardized enrichment score (NES), it could be observed that this pathway showed an upregulation trend in plaque tissues (NES = 0.54). This trend suggests that although the sample size is limited and the statistical significance is insufficient, tryptophan-related genes may be in an activated state as a whole in atherosclerotic plaques.

In lines 415:

All the analysis scripts used in this study have been stored on GitHub.

**3.Defects of data analysis method:**

**Box plots are required to visualize data distribution patterns, which can be created by adding data points or plotting violin-shaped histograms. The text fails to specify the exact methodology for scRNA batch correction (only mentioning the Harmony package), and should provide detailed parameter settings. Additionally, Figures 3A and 3B lack proper reference to hypothesis testing methodologies.**

Thank you for your suggestion, we modified figure 3A into a violin diagram, added the parameter description of harmony in the Method section of the manuscript, and marked the test methods used in the captions of figures 3A and B.

In lines 142-143:

Harmony was run with default parameters (θ = 2, λ = 1, σ = 0.1, block.size = 0.05, max.iter.cluster = 20, max.iter.harmony = 10).

In lines 249-254:

(A) The expression difference of six TRDEGs in carotid plaque and normal tissue, and the differential expression of all genes met significance thresholds: |log2FC| > 0.75 and p.value < 0.05. Statistical significance between groups was determined using the Wilcoxon rank-sum test. (B) Differences in immune cell infiltration proportions between carotid plaque and normal tissue were evaluated using the Wilcoxon rank-sum test. * P < 0.05, ** P < 0.01, *** P < 0.001, ns non-significant.

**Reviewer #3**

**1.The analysis of this work is based on a simple study (RNA-seq, GSE43292; scRNA-seq, GSE159677) with an imbalance of the patient numbers between these two datasets. Do the authors consider any artifacts of the analyses caused by inter-patient variants? If so, were any steps of the quality control at the experimental and/or analytical steps included? Please comment on it.**

Thank you for your suggestion, we acknowledge the reviewers' concern about the potential human influence resulting from the variability among patients in the datasets. To minimize this impact, we implemented several quality control steps at both the batch and single-cell levels. For the bulk RNA-seq dataset (GSE43292), we used the preprocessed and normalized data stored in GEO and applied DESeq2 for differential expression analysis to explain the variance between samples. For the single-cell RNA-seq dataset (GSE159677), We retain high-quality cells (nFeature_RNA > 200, nFeature_RNA < 6000, percent.mt < 10, and log10GenesPerUMI > 0.8), and apply normalization and scaling programs in Seurat to reduce technical noise. These steps ensure that our discovery is robust.

**2.Line 185: The authors selected 6 TRDEGs from a set of 50 genes without clarifying the selection criteria. Please provide more details about the strategy that indicated 6 TRDEGs were picked up.**

Thank you for your suggestion, we described in detail the method of TRDEGs screening in the revised manuscript.

In lines 183-188:

By analyzing the cross-relationship between DEGs and genes related to tryptophan metabolism, we prioritized the genes based on three criteria: (1) There was a significant difference in expression between carotid plaque and normal samples (adjusted p value < 0.05, log2FC > 0.75); (2) Included in the gene set related to tryptophan metabolism. Applying these standards, we ultimately identified six key TRDEGs -TPH1, MAOB, TDO2, KMO, KYNU and CYP1B1 - laying the foundation for subsequent analysis (Fig 2A).

**3.Line 196: The AUC value of 0.75 is however not optimal. This observation probably reflects the possibility of insufficiency of selected TRDEGs. Aligning with the previous concern, a detailed explanation of the selection criteria is needed. In addition, it is recommended that the authors construct an additional classifier to verify the findings computed from the model constructed by logistic regression.**

Thank you for your suggestion, we constructed four additional classifiers (GLM, LASSO, Random Forest, and SVM) to confirm the robustness of the TRDEG-based signature. All models achieved comparable performance (AUC 0.80–0.83) under cross-validation, demonstrating that the predictive value of the six TRDEGs is stable and not limited to a specific algorithm. These results have been added to the revised manuscript.

In lines 108-117:

To verify the robustness of the six-gene signature, we trained four supervised classifiers, including logistic regression (GLM), LASSO-regularized logistic regression, Random Forest, and support vector machine (SVM, radial kernel). All models were trained using the same six TRDEGs as predictors, and evaluated by stratified 5-fold cross-validation to avoid sampling bias. Model performance was assessed using (1) area under the ROC curve (AUC) for discrimination, (2) area under the precision–recall curve (PR-AUC) for performance under class imbalance, and (3) the Brier score to quantify probability calibration. All classifiers were implemented using the caret framework in R, ensuring identical data partitioning and evaluation procedures across models. ROC and PR curves were generated for visualization, and a summary performance table was created to compare classifier outputs.

In lines 201-210:

The logistic regression classifier achieved an AUC of 0.82, with a PR-AUC of 0.82, and a Brier score of 0.19, indicating good discrimination and acceptable probability calibration. To further verify that the predictive value of the six-gene panel was not model-dependent, three additional classifiers (LASSO, Random Forest, and SVM) were applied using the same cross-validation strategy. All models demonstrated comparable performance, with AUC values ranging from 0.80 to 0.83, PR-AUC values between 0.71 and 0.82, and Brier scores between 0.18 and 0.19 (Table 2). ROC and PR curves showed similar shapes across all four classifiers (Fig.2E-F), supporting that the six-gene signature provides consistent and reproducible predictive ability for distinguishing carotid plaque from normal tissue, independent of algorithm choice.

**Table 2. Performance metrics of four TRDEG-based models.**

| Model | AUC | PR_AUC | Brier |
| --- | --- | --- | --- |
| GLM | 0.82 | 0.82 | 0.19 |
| LASSO | 0.80 | 0.73 | 0.19 |
| RandomForest | 0.82 | 0.76 | 0.18 |
| SVM | 0.83 | 0.71 | 0.18 |

In lines 214-219:

(E) Receiver operating characteristic (ROC) curves of the four classification models (GLM, LASSO, Random Forest, and SVM) constructed using the six TRDEGs. Model performance was evaluated by stratified 5-fold cross-validation, and the area under the ROC curve (AUC) was calculated for each classifier. (F) Precision-recall (PR) curves and calibration results of the four TRDEG-based models. PR curves reflect classifier performance in imbalanced settings, while calibration curves evaluate the agreement between predicted and observed probabilities.

**4.Lines 219 - 221: The description related to Figure 3B escaped me. If I interpreted the plot correctly, the proportion of M0 macrophages showed an increase in carotid plaques, whereas a decrease in the proportions of CD8+ T cells, activated NK cells, monocytes, and activated DCs was detected.**

Thank you for your suggestion, we corrected these two writing errors in the revised manuscript.

In lines 234-238:

The results of CIBERSORT indicate that the proportions of memory B cells, activated CD4^+^ memory T cells, and M0 macrophages in carotid plaque tissues are significantly increased (Wilcox test, p value < 0.05), while the proportions of CD8^+^ T cells, activated NK cells, monocytes, and activated dendritic cells are significantly decreased (Wilcox test, p value < 0.05，Fig 3B).

**5.Line 230: It is not clear to me about the immune microenvironment mentioned here. Please elaborate more about this immune microenvironment associated with Tryptophan-related pathways triggered by TRDEGs in the Discussion.**

Thank you for your suggestion, we added immune microenvironment associated with Tryptophan-related pathways triggered by TRDEGs in the Discussion.

In lines 372-379:

Atherosclerotic plaques typically present a chronic inflammatory microenvironment characterized by infiltration of macrophages, T cells and dendritic cells. Metabolic reprogramming of the kynuurine pathway can be achieved by consuming local tryptophan and generating various immunomodulatory metabolites (such as Kyn and 3-HK). It triggers T-cell exhaustion and macrophage phenotypic polarization, thereby driving immunosuppression and the persistence of chronic inflammation. Therefore, the abnormal expression of TRDEGs may be a key molecular hub connecting metabolic imbalance and immune disorder, providing a new explanatory framework for us to understand the "metabolism-immune crosinterference" in atherosclerosis.

**6.Line 251: It is not clear to me why, for example, KYNU possesses fewer degrees than other nodes (i.e., KMO, TDO2, and TPH1) in the network; however, it is shown in the network that a high edge connectivity of the node KYNU is demonstrated. Furthermore, it is not clear how “closeness” is calculated here. In principle, the edge connectivity (or correlation coefficients) has to be calculated based on statistical significance (e.g., Pearson correlation). It is therefore not understandable to me why the value can be 1. Nevertheless, please provide more details on how parameters related to the graph network are computed.**

Thank you for your suggestion, we will explain our calculation method to you.The PPI network for the TRDEGs was constructed using interaction data from the STRING database. We imported the interaction table into R and generated an undirected network using the igraph package. The network was treated as unweighted, meaning that all edges were considered equal. Network centrality measures were calculated as follows: Degree centrality: the number of direct connections of a node within the network. Betweenness centrality: the number of shortest paths passing through a node, reflecting its role in connecting other nodes. Closeness centrality: the inverse of the sum of shortest path lengths from a node to all other nodes, normalized by igraph. These centrality measures describe the topological properties of nodes within the subset of TRDEGs, rather than the full interactome. Differences between nodes (e.g., KYNU vs KMO) reflect their connectivity among the selected genes. Edge connectivity in this analysis is based solely on the presence of edges and does not incorporate statistical significance from STRING scores. The network visualization and centrality metrics were used to identify hub genes potentially important for carotid plaque formation.For more detailed calculation methods, you can refer to the code we provide.

**7.Line 253: Figure 3A seems to be inconsistent with the text mentioned here. How bar graphs can display the interactions across proteins.**

Thank you for your suggestion, we corrected this writing error in the revised manuscript and added the description of the filtering conditions for PPI interactions in the “String”.

In lines 271-274:

The light blue lines indicate that there are "Known Interactions" between proteins from "from curated databases".It can be observed from figure 4A that the known interactions exist among four pairs of proteins: KYNU - KMO, KYNU - TDO2, KMO - CYP1B1, and TDO2 - TPH1.

**8.Line 284: In Figure 5D, I did observe a noticeable variation across patients marked in the same groups (AC versus PA), resonating with my comment #1. Since variations exist, a proper statistical test is required here.**

Thank you for your suggestion. I think what you mean is that statistical tests should be added in figure 5E. In the revised manuscript, we reanalyzed the cell type composition using the speckle R package and applied benjamin hochberg FDR correction. The updated results (new Figures 5E and S5 tables) show that although no cell type achieved fdr adjustment significance due to the variability among patients and the limited sample size (3 vs 3), the proportion trend remains biologically consistent with an increase in immune cell participation and a decrease in the number of structural cells in carotid artery plaques.

In lines 308-317:

We next assessed cell-type composition in carotid plaques using scRNA-seq. Although no cell type reached statistical significance after FDR correction (Fig. 5E; S5 table), endothelial cells, fibroblasts, and smooth muscle cells showed decreasing proportions in plaques, whereas macrophages and dendritic cells exhibited an increasing trend. These compositional shifts provide a plausible explanation for TRDEG expression patterns. Five genes (KMO, TDO2, CYP1B1, KYNU, and MAOB) were consistent between bulk and single-cell data, while TPH1 was elevated in fibroblasts and smooth muscle cells but downregulated in bulk tissue, likely due to the reduced abundance of these cell types in plaques. Together, these findings indicate that TRDEG heterogeneity is shaped by both transcriptional changes and cell-type proportion shifts within the plaque microenvironment.

**9.Line 289: Once again, I cannot link Figure 4E to the text here. Figure 4E describes the pathway enrichment, instead of the proportion of cells.**

Thank you for your suggestion, we corrected this writing errors in the revised manuscript.

In lines 308-311:

We next assessed cell-type composition in carotid plaques using scRNA-seq. Although no cell type reached statistical significance after FDR correction (Fig. 5E; S5 table), endothelial cells, fibroblasts, and smooth muscle cells showed decreasing proportions in plaques, whereas macrophages and dendritic cells exhibited an increasing trend.
