# Supplementary material for: Identification and characterization of tryptophan metabolism-related genes in carotid artery plaques
Source: PLoS One. 2026 Feb 2;21(2):e0341122. doi: 10.1371/journal.pone.0341122 (PMC12863520; doi:10.1371/journal.pone.0341122)
Supplement: Attachment — Submitted filename: Response_to_Reviewers_auresp_2.docx [file pone.0341122.s008.docx]

**Academic editor**

**Although this study offers initial insight into tryptophan-related gene changes in carotid atherosclerotic plaques, several limitations should be noted. The analysis relies on a single bulk RNA-seq dataset, and the absence of additional human or animal cohorts limits the robustness of the differential gene results. The enrichment of the tryptophan metabolism pathway did not reach statistical significance, likely due to limited sample size. Immune infiltration estimates and single-cell annotations were derived computationally without experimental validation, which may introduce bias. Additionally, no functional assays were performed to confirm the roles of the identified genes in atherosclerosis. These points should be acknowledged in the manuscript so that the readership will consider them when interpreting the findings.**

Thank you for your suggestion. We sincerely appreciate the editor’s constructive summary of the study’s limitations. We fully agree that transparently acknowledging these constraints is essential for the readership to properly interpret the scientific validity and scope of our findings. As suggested, we have added a dedicated "Limitations" paragraph at the end of the Discussion section (Lines 380-393). In this new section, we explicitly discuss the reliance on a single dataset, the sample size constraints affecting statistical significance in pathway enrichment, the computational nature of the immune analysis, and the lack of wet-lab validation.

In lines 380-393:

Despite the initial insights offered by this study regarding tryptophan-related gene changes in carotid atherosclerotic plaques, several limitations should be acknowledged. First, our analysis relied on a single bulk RNA-seq dataset. The absence of validation in additional independent human cohorts or animal models limits the robustness and generalizability of the identified differential gene signatures. Second, although we observed biological trends relevant to the study's hypothesis, the enrichment of the tryptophan metabolism pathway did not reach statistical significance, which is likely attributable to the limited sample size of the current dataset. Third, the immune infiltration estimates and single-cell type annotations were derived exclusively through computational algorithms. While these methods provide a systems-level perspective, the lack of experimental corroboration may introduce potential bioinformatic biases. Finally, this study remains descriptive in nature, as no functional assays were performed to mechanistically confirm the specific roles of the identified genes in the progression of atherosclerosis. Consequently, these findings should be interpreted with caution, and future studies incorporating larger sample sizes and experimental validation are warranted to verify these preliminary results.
